# Transcriptome Analysis Reveals a Promotion of Carotenoid Production by Copper Ions in Recombinant *Saccharomyces cerevisiae*

**DOI:** 10.3390/microorganisms9020233

**Published:** 2021-01-23

**Authors:** Buli Su, Anzhang Li, Ming-Rong Deng, Honghui Zhu

**Affiliations:** State Key Laboratory of Applied Microbiology Southern China, Guangdong Provincial Key Laboratory of Microbial Culture Collection and Application, Guangdong Microbial Culture Collection Center (GDMCC), Guangdong Institute of Microbiology, Guangdong Academy of Sciences, Guangzhou 510070, China; bolysu@hotmail.com (B.S.); liaz@gdim.cn (A.L.); dengmr@gdim.cn (M.-R.D.)

**Keywords:** *Saccharomyces cerevisiae*, carotenoids, copper, zinc, *ACE1*, *ADY2*

## Abstract

We previously constructed a *Saccharomyces cerevisiae* carotenoid producer BL03-D-4 which produced much more carotenoid in YPM (modified YPD) media than YPD media. In this study, the impacts of nutritional components on carotenoid accumulation of BL03-D-4 were investigated. When using YPM media, the carotenoid yield was increased 10-fold compared to using the YPD media. To elucidate the hidden mechanism, a transcriptome analysis was performed and showed that 464 genes changed significantly in YPM media. Furthermore, inspired by the differential gene expression analysis which indicated that *ADY2*, *HES1*, and *CUP1* showed the most remarkable changes, we found that the improvement of carotenoid accumulation in YPM media was mainly due to the copper ions, since supplementation of 0.08 mM CuSO_4_ in YPD media could increase carotenoid yield 9.2-fold. Reverse engineering of target genes was performed and carotenoid yield could be increased 6.4-fold in YPD media through overexpression of *ACE1*. The present study revealed for the first time the prominent promotion of carotenoid yield by copper ions in engineered *S. cerevisiae* and provided a new target *ACE1* for genetic engineering of *S. cerevisiae* for the bioproduction of carotenoids.

## 1. Introduction

Carotenoids are a large family of colored compounds naturally present in plants, algae, fungi, and bacteria. These compounds are known antioxidants that have a potentially beneficial effect on human health and may be used for nutrition, food, and cosmetic applications [1]. Currently, chemical synthesis and natural extraction from plants or algae are the major processes for carotenoid production in industry. However, chemical synthesis is very difficult due to the structural complexity of most carotenoids, and natural extraction based on solvent from plants and algae is confronted with a lot of challenges such as unpredictable feedstock availability, organic pollutants, and low yields [2]. Thus, the biosynthesis of certain carotenoids through homologous or heterologous microorganisms is becoming more and more attractive [3].

With the rapid development of metabolic engineering and synthetic biology, engineering microorganisms for the heterologous production of carotenoids has become a more sustainable process. Thus, there have been extensive studies to engineer microbial cell factories for the production of carotenoids [4]. The majority of these efforts were focused on the optimization of endogenous pathways, the introduction of heterologous genes to boost metabolic flux, and balancing cofactors [5]. Multiple strategies have been adopted to enhance the production of carotenoids in *S. cerevisiae*, such as boosting the metabolic flux towards the mevalonate (MVA) pathway through overexpression of truncated 3-hydroxy-3-methylglutaryl coenzyme A reductase (tHMG1) [6], increasing the supply of precursors (acetyl-CoA) through downregulation of squalene synthase (Erg9) or overexpression of acetaldehyde dehydrogenase (ALD6) and acetyl-CoA synthetase (ACS) [7,8]. Besides, strengthening the pathways such as the TCA cycle and pentose phosphate (PPP) pathway could increase the cofactor supply which carotenoid biosynthesis needs [9]. However, according to previous reports, little was demonstrated about the impact of nutritional components on carotenoids yield of recombinant strains, except one case that carotenoid production increased 7-fold using fructose as the carbon source in engineered *E. coli* [10].

Previously, we engineered a synthetic pathway for carotenoid biosynthesis (using lycopene as the model carotenoid) in *S. cerevisiae* and gained a higher production using the YPM media [11,12]. In this study, we aim to determine the impacts of nutritional components on the carotenoid yield of BL03-D-4 generated from our prior work, and then attempt to uncover the potential mechanisms of the promoted carotenoid production associated with supplementation of nutritional components by transcriptome analysis.

## 2. Materials and Methods

### 2.1. Strains and Cultivation

The original strain, engineered strains, and plasmids used in this study are listed in Table S1. Primers are provided in Table S2. Cloning and maintenance of plasmids were carried out in *E. coli* DH5α. Phanta Max Super-Fidelity DNA Polymerase was obtained from Vazyme Biotech Co., Ltd. (Vazyme, Nanjing, Jiangsu, China). HiPure Gel Pure Micro Kit and HiPure Plasmid Plus Micro Kit were purchased from Magen (Guangzhou, Guangdong, China). Tryptone and yeast extraction (LOT: 2315359-02 and 2665431-02) were acquired from Oxoid (Basingstoke, Hampshire, UK) and Angel (Yichang, Hubei, China) (FM802, LOT: 2018082210C9). Other compounds unless specifically mentioned were of reagent grade.

### 2.2. Genetic Manipulation

CRISPR/Cpf1 system was applied for *ADY2* and *ACE1* deletion, and overexpression of *HES1*, *ACE1*, *CUP1*, and *SOD1* by genomic integration. The pHCas9M-gRNA plasmid (Molecular Cloud: MC_0000739) was used for Cpf1-based genetic modifications by replacing the sgRNA and Cas9 on plasmid pHCas9M-gRNA with Cpf1 and crRNA from plasmid pCSN067 (Addgene#101748) [13]. The homologous arms of target genes under the yeast promoter *Cit1* or *TEF2* and native terminators were used as donor DNA. The edited plasmid including the guide RNA that targeted the chromosomal site was cotransformed into yeast cells with the donor DNA according to previous protocol [14]. Cells were screened on the YPD plate supplemented with 400 µg mL^−1^ G418. Positive colonies were confirmed by diagnostic PCR.

### 2.3. Shake-Flask Fermentation and Growth Determination

For cultivation, a single colony was picked up from a fresh YPD plate and transferred to 5 mL of YPD medium. Strains were cultured overnight at 200 rpm and 30 °C, 1 mL seed culture was inoculated into a 250 mL shake-flask with 50 mL YPD medium, modified YPD media (YPM), YPD media supplemented with different chemicals, YPX media or YPX media supplemented with 0.08 mM copper ions, and defined media (DM) or DM media supplemented with 0.08 mM different ions to an optical density (OD_600_) of 0.05, and then allowed to incubate at 30 °C for 72 to 120 h. After incubation, cultures were taken and analyzed for biomass and carotenoid content. YPD1, YPD2, and YPD3 media contained 20 g L^−1^ tryptone, 20 g L^−1^ glucose, and 10 g L^−1^ different yeast extraction (LOTs of 2665431-02, 2315359-02, and 2018082210C9, respectively). YPX media contained 10 g L^−1^ yeast extraction (YPD2), 20 g L^−1^ tryptone, 30 g L^−1^ xylose, and 10 g L^−1^ glucose. DM media contained 6.7 g L^−1^ YNB, 1.29 g L^−1^ DO-supplement, and 20 g L^−1^ glucose. YPM media contained 10 g L^−1^ yeast extraction (FM802, LOT: 2018082210C9), 20 g L^−1^ tryptone, 20 g L^−1^ glucose, salt (10 g L^−1^ KH_2_PO_4_, 2.5 g L^−1^ MgSO_4_, 3.5 g L^−1^ K_2_SO_4_, 0.25 g L^−1^ Na_2_SO_4_), and 1 mL trace metal solution (TMS) described in previous literature [12]. Growth was determined according to the OD_600_ value and correlated to cell dry weight (CDW) by the CDW/OD_600_ standard curve (y = 0.184x + 0.891 (x is OD_600_, y is CDW, R^2^ = 0.992)).

### 2.4. Analytical Methods

Carotenoids were extracted using the method described in our previous work [11]. Briefly, strains cultures were centrifuged, washed, resuspended in 3 mol L^−1^ HCl boiling for 4 min, and cooled in an ice bath for 3 min. Then cell debris was washed twice, resuspended in acetone, ultrasonic extracted, and followed by centrifugation. The acetone supernatant was used for measuring total carotenoid by using a UV–VIS spectrometer (PerkinElmer Lambda 45, Waltham, MA, USA) at 470 nm. The extinction coefficient was adopted in acetone using an absorbance 1% 1 cm of 3450 [12]. Copper concentration was measured using the Copper (Cu) Colorimetric Assay Kit (Elabscience Biotechnology Co., Ltd., Wuhan, Hubei, China).

### 2.5. Transcriptome Analysis

Total RNA of different yeast cells was isolated after about 24 h cultivation using the HiPure Yeast RNA Kit (Magen, Guangzhou, Guangdong, China) according to the manufacturer’s protocol. Transcriptome analysis was performed by Majorbio (Shanghai Majorbio Bio-pharm Technology Co., Ltd.) through RNA sequencing. Briefly, the RNA quality was determined by 2100 Bioanalyser (Agilent, Santa Clara, CA, USA) and quantified using the ND-2000 (NanoDrop Technologies, Tokyo, Japan). The RNA-seq transcriptome library was prepared following the TruSeqTM RNA sample preparation kit from Illumina (San Diego, CA, USA). Messenger RNA was isolated according to polyA selection method by oligo(dT) beads and then fragmented by fragmentation buffer. Secondly double-stranded cDNA was synthesized using a SuperScript double-stranded cDNA synthesis kit (Invitrogen, Carlsbad, CA, USA) with random hexamer primers (Illumina). Then the synthesized cDNA was subjected to end-repair, phosphorylation, and “A” base addition according to Illumina’s library construction protocol. All raw data for RNA-seq was deposited into NCBI (GEO accession number GSE164229). Transcriptome data analysis is provided in Table S3. The quantitative analyses of gene expression were performed using the Transcripts Per Million (TPM) method by RSEM for each sample. The data were analyzed on the free online platform of the Majorbio Cloud Platform (www.majorbio.com).

### 2.6. Quantitative PCR

The total RNA was extracted from each yeast strain cultivated in YPD2 media for 24 h using the above kit, referencing the manual of application. The RNA samples were reversely transcribed using HiScript II Q RT SuperMix for qPCR (+gDNA wiper) Kit (Vazyme, Nanjing, Jiangsu, China). Quantitative PCR was performed using ChamQ Universal SYBR qPCR Master Mix (Vazyme, China) on a QuantStudio 6 Flex Real-Time PCR System (Life Technologies, Carlsbad, CA, USA). The internal control gene *ACT1* was chosen to normalize the different samples and the relative gene expression analysis was performed using the 2^−ΔΔ*C*T^ method.

## 3. Results and Discussion

### 3.1. Significant Promotion of Carotenoid Accumulation is Presented in a Modified YPD Medium

**Figure 1:**
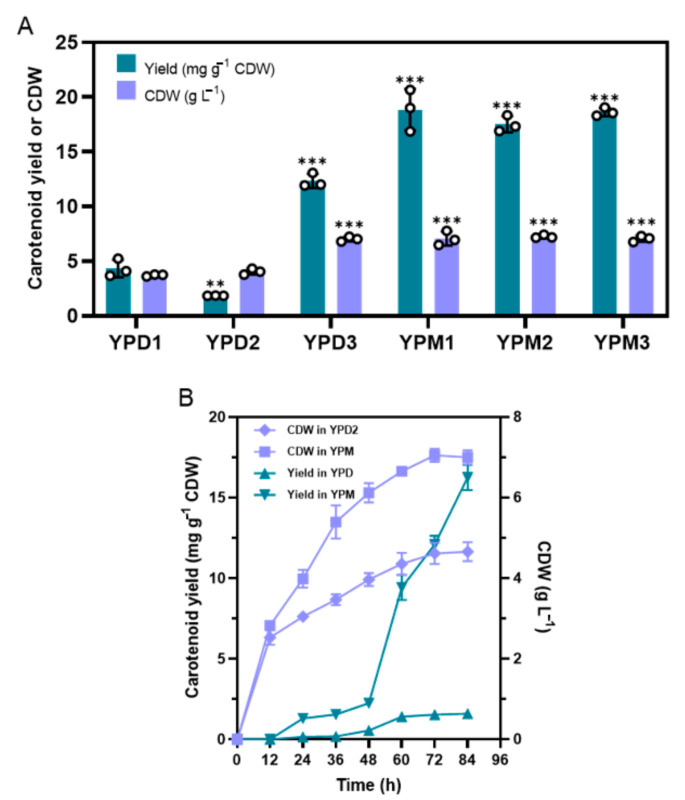
Carotenoid yields and cell masses when BL03-D-4 was cultivated for 96 h in YPD media with different yeast extractions or modified YPD (YPM) media (**A**). Time-courses of carotenoid fermentation in YPD2 and YPM media (**B**). YPD1, YPD media with yeast extraction of LOT: 2665431-02. YPD2; YPD media with yeast extraction of LOT: 2315359-02. YPD3; and YPD media with yeast extraction from Angel (FM802, LOT: 2018082210C9). YPM1, YPM2, and YPM3 represented three replicates using YPM media along with YPD1, YPD2, and YPD3, respectively. The values are the means and standard deviations of three biological replicates (small circles). **: significantly different based on two-tailed Student’s *t*-test (*p* < 0.01); ***: significantly different based on two-tailed Student’s *t*-test (*p* < 0.001).

In our experimental experience, we found that the carotenoid yield in engineered *S. cerevisiae* was fluctuant using different lots of yeast extraction and the engineered strains could gain a stably higher carotenoid in the YPM media [11]. To clarify the impacts of nutritional components of the YPM medium on carotenoid yield, we compared the biomasses and carotenoid accumulations of BL03-D-4 growing on different YPD and YPM media. As illustrated in Figure 1A, obvious different growth and carotenoid accumulations were obtained when strains were cultured in different media. Both manufacturers (Oxoid and Angel) and lots of yeast extraction had significant effects on carotenoid accumulation indicating that there were different compounds in these different yeast extractions (Figure S1). When cultured in the YPM media, the final carotenoid yield was 18.8 mg g^−1^ CDW, which was distinctly improved and over 10-fold higher compared to the yield of 1.85 mg g^−1^ CDW in the YPD2 medium (Figure 1A). The maximum biomass reached 4.1 g L^−1^ of culture in the YPD2 media. When using YPM, the final cell mass could reach 7.0 g L^−1^, which was 1.7-fold of that in the YPD2 media. The time-courses of carotenoid fermentation in YPD2 and YPM media were also measured (Figure 1B); the growth curves and yield curves represented obvious differences. These results indicated that some substances in the YPM medium could promote cell growth and carotenoid biosynthesis. However, the reason behind this manner has not yet been known to necessitate further studies. For the sake of gaining a deeper understanding of the mechanisms, comprehensive analyses such as gene expression can be executed.

### 3.2. Transcriptome Analysis Reveals Multiple Differentially Expressed Genes

In this study, we investigated and analyzed the comprehensive transcriptome data of BL03-D-4 in YPD2 and YPM media. Strains typically reach a steady state level of transcription in the stationary phases [11]. Thus, gene expression patterns of BL03-D-4 in stationary phases (about 24 h) cultured with these two different media were compared using transcriptome sequencing. The results of RNA-seq data analysis are supplemented in Table S3 and showed that 464 genes changed their expression levels significantly (*p* < 0.05; |Log2FC| ≥ 1). Among aforesaid genes, 203 genes were upregulated and 261 were downregulated compared to using the YPD media.

**Figure 2:**
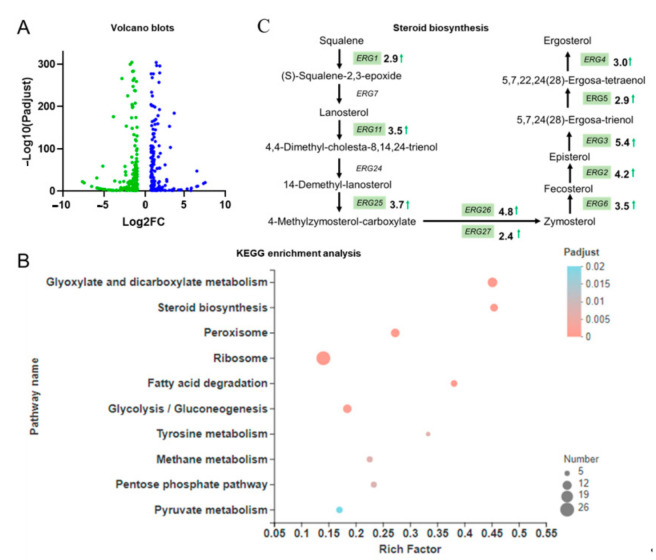
The analysis of differentially expressed genes (DEGs). (**A**) Volcano blots to show significant DEGs. Dispersion graph of the −log10(P adjusted) (*y*-axis) against the log2(FC) (*x*-axis). Green dots and blue dots represent genes that were significantly downregulated and upregulated, respectively, in YPM media for 24 h. (**B**) KEGG pathway enrichment of upregulated genes in the YPM media for 24 h; (**C**) The expression pattern of genes in the steroid biosynthesis pathway in the YPM media; green arrows represented up-regulation and bolded numbers represented the fold of up-regulation.

KEGG pathway analysis was performed to search genes that might play key roles in the promotion of carotenoid accumulation. KEGG pathway analysis showed that genes in the glyoxylate and dicarboxylate metabolism, steroid biosynthesis, fatty acid degradation, glycolysis/gluconeogenesis, and PPP pathways changed significantly (Figure 2). These results were not surprising since the glycolysis and PPP pathways could provide energy for cell growth and cofactors for carotenoid biosynthesis [15]. Facilitation of steroid and fatty acid for carotenoid accumulation has been well studied previously [16], and interestingly these pathways were also shown to be clustered in this study. In particular, as shown in Figure 2C, most genes in the ergosterol biosynthetic pathway (ERG) containing *ERG1*, *ERG11*, *ERG25*, *ERG26*, *ERG27*, *ERG6*, *ERG2*, *ERG3*, *ERG5*, and *ERG4* were significantly upregulated in the YPM media. This gene expression pattern of the ergosterol biosynthetic pathway was similar to a previous study which indicated that deletion of the acetate transporter gene *ADY2* could improve the tolerance of *S. cerevisiae* against multiple stresses including acetic acid, ethanol, and hydrogen peroxide stresses [17]. The cell membrane of microorganisms contained a variety of sterols including ergosterol, fecosterol, and zymosterol [18]. Ergosterol played a key role in maintaining yeast membranes stabilization and was involved in carotenoid accumulation [16]. Besides, ergosterol biosynthesis showed facilitation for growth under ethanol stress [19]. Therefore, optimization of the sterol composition was an effective method of regulating membrane lipid homeostasis through changing the content of particular sterols and manipulating the sterol categories present [20,21]. Furthermore, sterol content has been reformed through overexpression of *Erg3*, *Erg2*, and *Erg5* in sterol biosynthetic pathways [22]. However, based on the literature, the main factors that regulate *ERG* gene expression are the levels of unsaturated fatty acids, oxygen, and osmotic stress, and *ERG* gene expression is coordinated by sterol regulatory element (SRE)-binding proteins Upc2 and Ecm22, the heme-binding protein Hap1, and the repressor factors Rox1 and Mot3 [23].

We were interested in the significantly changed genes since these genes would provide indirect clues of the mechanisms that caused different carotenoid yields in different media. Among dramatically upregulated genes involved in steroid biosynthesis, *HES1* encoded a protein that was implicated in the regulation of ergosterol biosynthesis, and it was one of the sterol transporters which transported sterol from the endoplasmic reticulum (ER) to the mitochondria [24]. On the other hand, acetate transporter gene *ADY2*, which was responsive for zinc ions, was downregulated 8-fold. Besides, the *CUP1-2* gene, which encoded copper metallothionein, was upregulated about 94-fold. It is therefore of interest to study whether supplementation of zinc or/and copper ions can improve the carotenoid yield of BL03-D-4.

### 3.3. Copper Ions Play a Key Role in the Carotenoid Accumulation of BL03-D-4

**Figure 3:**
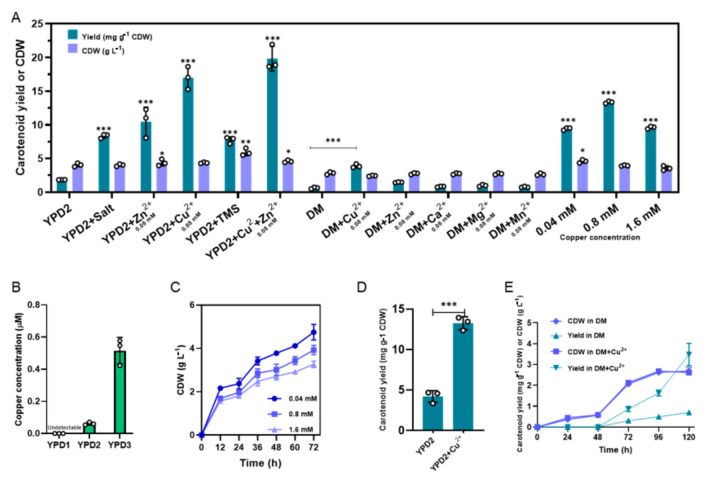
Carotenoid yields and cell masses in the YPD2 or defined media (DM) media supplement with different chemicals or different concentrations of copper (**A**). Copper concentrations in different yeast extractions (**B**). Growth curves of BL03-D-4 in YPD2 media supplement with different concentrations of copper (**C**). Carotenoid yields of SC106 in YPD2 media and YPD2 supplemented with 0.08 mM copper (**D**). Time-courses of carotenoid fermentation in DM and DM supplemented with 0.08 mM copper (**E**). The values are the means and standard deviations of three biological replicates (small circles). *: significantly different based on two-tailed Student’s *t*-test (*p* < 0.05); **: significantly different based on two-tailed Student’s *t*-test (*p* < 0.01); ***: significantly different based on two-tailed Student’s *t*-test (*p* < 0.001).

To find out the key factor of the promotion of carotenoid accumulation, YPD media supplemented with different chemicals were used for carotenoid fermentation. As shown in Figure 3A, salt (10 g L^−1^ KH_2_PO_4_, 2.5 g L^−1^ MgSO_4_, 3.5 g L^−1^ K_2_SO_4_, 0.25 g L^−1^ Na_2_SO_4_) and TMS (trace metal solution) had a promoting effect on carotenoid production. Better results were observed in the YPD2 medium supplemented with zinc (0.08 mM) and copper ions (0.08 mM). Especially, supplementation of copper ions could account for 90% of the improvement, and even gain lower biomass compared to using the YPM media. Furthermore, different concentrations of copper presented different promotions, while a very high concentration of copper was toxic to cells (Figure 3C). It is worth mentioning that simultaneous supplementation of zinc and copper ions could increase the carotenoid yield by 9.7-fold to 19.8 mg g^−1^ CDW which exceeded the promotion of using the YPM media. It was likely that copper ions also influenced carotenoid biosynthesis by upregulating genes in the sterol biosynthetic pathway since there were no other significantly changed genes in any pathway like ergosterol (Figure 2C). Figure 3 shows that those up to 0.08 mM copper-added media displayed good growth and carotenoid yield of BL03-D-4, while those with up to 1.6 mM copper in the media caused side effects. These findings were quite similar to the results of a previous study in microalgae which showed that the addition of a suitable concentration of copper in the media stimulated microalgal growth, as well as lutein content [25]. We also measured the copper concentration of different yeast extractions and found that there were significant differences in copper concentration of these yeast extractions (Figure 3B). This result added to the growing body of evidence that copper ions are responsible for the promotion of carotenoid production. To verify whether the facilitation of copper ions was general, DM media and another carotenoid producer SC106 were used. As shown in Figure 3, Figure S1 (shake-flask fermentations), and Figure S1B,C (spot tests), copper ions could also facilitate carotenoid accumulation in DM media, although the carotenoid yield and cell masses were very low. Interestingly, the carotenoid yield of SC106 was increased 3.2-fold from xylose-glucose mixtures in YPD2 media supplemented with 0.08 mM copper ions.

### 3.4. ACE1 Overexpression Remarkably Improves Carotenoid Accumulation of BL03-D-4

**Figure 4:**
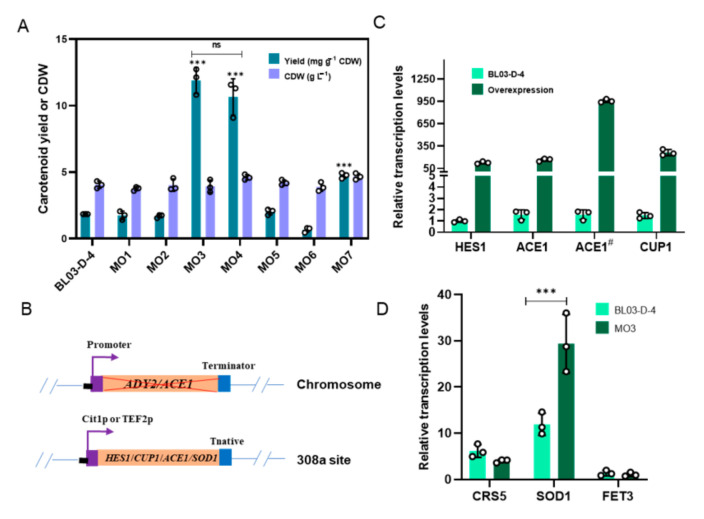
Reverse engineering of target gene mutation and overexpression for carotenoid accumulation in the YPD2 media for 96 h. MO1, deletion of *ADY2*; MO2, overexpression of *HES1*; MO3, overexpression of *ACE1* under *Cit1* promoter; MO4, overexpression of *ACE1* under *TEF2* promoter; MO5, overexpression of *CUP1*; MO6, deletion of *ACE1*; MO7, overexpression of *SOD1* (**A**). Schematic explaining gene mutation and overexpression; purple arrows represented promoter (**B**). Relative transcription levels of *HES1*, *ACE1*, and *CUP1* overexpressed under *Cit1* promoter compared to BL03-D-4; ACE1^#^, *ACE1* overexpressed under *TEF2* promoter; strains were cultivated in YPD2 media for 24 h (**C**). Relative transcription levels of *CRS5*, *SOD1*, and *FET3* in MO3 compared to BL03-D-4 in YPD2 media for 24 h (**D**). Data represent the means and standard deviations of three biological replicates (small circles). ***: significantly different based on two-tailed Student’s *t*-test (*p* < 0.001), ns: not significant (*p* > 0.05).

To determine whether the selected targets were conducive to the promotion of carotenoid accumulation, gene deletion and gene overexpression were conducted in BL03-D-4, followed by the analysis of growth and carotenoid yield in YPD2 media. A single-gene knockout mutant of *ADY2* and *ACE1* were constructed using the CRISPR/Cpf1 system. The *HES1, ACE1*, *CUP1*, and *SOD1*-overexpressed strains were obtained, respectively, through integrating the overexpression cassettes in the *308a* site [26]. As shown in Figure 4, the deletion of *ADY2* and overexpression of *HES1* did not increase the carotenoid accumulation. Interestingly, *ACE1* which is the activating factor of *CUP1* overexpressed strain MO3 can accumulate 6.4-fold higher carotenoid than the control strain, and this amount of increase accounts for over 60% of the carotenoid yield when using YPM media. However, no significant difference was observed in overexpression of *ACE1* under the control of a stronger promoter *TEF2*. Since the facilitation partially accounts for the carotenoid promotion, these results prompted the speculation that the promotion of carotenoid accumulation might be regulated by a multi-gene which was responsible for zinc and copper ions. We also overexpressed the *CUP1* gene; however, no facilitation was observed (Figure 4). We sequenced the genome of BL03-D-4 and the alterations in the copy numbers of the *CUP* genes were not observed. The fastq DNA-seq data were deposited in the Genome Sequence Archive (GSA) server at the BIG Data Center in Beijing Institute of Genomics (GSA accession No. CRA003704). It is worth mentioning that *ACE1* also activates in response to copper other genes such as *CRS5*, *SOD1*, and *FET3*/*FTR1* [27]. We have quantified the amount of these genes in MO3 using qPCR, and *SOD1* represented remarkable upregulation. Furthermore, *SOD1* was also overexpressed, and carotenoid yield increased 2.6-fold. This may provide clues to uncover the molecular mechanism for carotenoid promotion. The dataset generated during the current study are available in Table S4.

To the best of our knowledge, our study demonstrated for the first time that the addition of copper and/or zinc ions had a significant promotion of carotenoid accumulation in *S. cerevisiae*, and the simultaneous supplementation of zinc and copper ions had a synergetic effect on carotenoid accumulation.

## Data Availability

All raw data for RNA-seq were deposited into NCBI (GEO accession number GSE164229) and fastq DNA-seq data of BL03-D-4 were deposited in the Genome Sequence Archive (GSA) server at the BIG Data Center in Beijing Institute of Genomics (GSA accession No. CRA003704).

## Supplementary Materials

The following are available online at https://www.mdpi.com/2076-2607/9/2/233/s1, Figure S1: Shake-flask fermentations and normalized spot tests, Table S1: Strains and plasmids used in this study, Table S2: Primers used in this study, Table S3: RNA-seq data analysis, Table S4: Raw data used in this study.
