# Supplementary material for: Transcriptome Analysis Reveals a Promotion of Carotenoid Production by Copper Ions in Recombinant Saccharomyces cerevisiae
Source: Microorganisms. 2021 Jan 23;9(2):233. doi: 10.3390/microorganisms9020233 (PMC7912134; doi:10.3390/microorganisms9020233)
Supplement: Supplementary file 1 [file microorganisms-09-00233-s001.zip › Table S1.docx]

**Table S1.** Strains and plasmids used in this study.

| Strain/plasmid | Description | Source |
| --- | --- | --- |
| *Strains* |  |  |
| DH5ɑ | *supE44 ΔlacU169 (φ80 lacZΔM15) hsdR17 recA1 endA1 gyrA96 thi-1 relA1* | Invitrogen |
| BL03-D-4  SC106 | BY4742, Δ*Gal80⸬* *P_HSP26_-CrtB-T_ADH1_-P_HSP26_-CrtI-T_GPM1_-P_HSP26_-CrtE-T_CYC1，_Δ416d⸬* *P_Cit1_-tHMGR-T_Guo,_ ΔAld6*  BY4742, Δ*Gal80⸬* *P_HSP26_-CrtB-T_ADH1_-P_HSP26_-CrtI-T_GPM1_-P_HSP26_-CrtE-T_CYC1，_Δ416d ⸬ P_Cit1_-tHMGR-T_Guo,_ ΔAld6*, *Pxk ⸬* *P_EFT1_-XYL1m-Tsyn-P_PGK1_-XYL2-Tsyn-P_HSP82_*_,_*Δ720a ⸬* *P_HSP104_-xPk-Tsyn-P_SSA1_-PTA-Tsyn*,*ΔPho13*,*ΔP_Gal2_ ⸬* *P_SSA1_-Gal2m*,*Δ911b ⸬ His3-Leu2-Ura3* | [12]  [12] |
| MO1  MO2 | BL03-D-4, *ΔADY2*  BL03-D-4, *Δ308a ⸬* *P_Cit1_-HES1-T_native_* | This study  This study |
| MO3  MO4  MO5  MO6  MO7 | BL03-D-4, *Δ308a ⸬* *P_Cit1_-ACE1-T_native_*  BL03-D-4, *Δ308a ⸬* *P_TEF2_-ACE1-T_native_*  BL03-D-4, *Δ308a ⸬* *P_Cit1_-CUP1-T_native_*  BL03-D-4, *ΔACE1*  BL03-D-4, *Δ308a ⸬* *P_Cit1_-SOD1-T_native_* | This study  This study  This study  This study  This study |
| *Plasmids* |  |  |
| pHCas9-gRNA  pCSN067  pCpf1-ADY2  pCpf1-ACE1  pCpf1-308a | pBR322-derived vector, including HCas9, KanMX, gRNA and 2μ ori, ampicillin resistant  Plasmid for Cpf1-based genome editing  Genome editing plasmid targeted to the *ADY2* loci  Genome editing plasmid targeted to the *ACE1* loci  Genome editing plasmid targeted to the *308a* loci | [11]  [13]  This study  This study  This study |
