# Supplementary material for: Transcriptome Analysis Reveals a Promotion of Carotenoid Production by Copper Ions in Recombinant Saccharomyces cerevisiae
Source: Microorganisms. 2021 Jan 23;9(2):233. doi: 10.3390/microorganisms9020233 (PMC7912134; doi:10.3390/microorganisms9020233)
Supplement: Supplementary file 1 [file microorganisms-09-00233-s001.zip › Table S2.docx]

**Table S2.** Primers used in this study. Homologous overhang-nucleotides (underlined); genomic target (red, bold).

| Primers | Sequence (5’-3’) |  |
| --- | --- | --- |
| Cpf1-UP-F  Cpf1-UP-R  Cpf1-DOWN-ADY2-F  Cpf1-DOWN-ACE1-F  Cpf1-DOWN-R  ADY2-UP-F  ADY2-UP-R  ADY2-DOWN-F  ADY2-DOWN-R  ACE1-UP-F  ACE1-UP-R  ACE1-DOWN-F  ACE1-DOWN-R  Cpf1-DOWN-308-F  ARS308a-F  Cit1-F  Cit1-R  HES1-F  HES1-R  CUP1-F  CUP1-R  SOD1-F  SOD1-R  ARS308a-R  308-CHECK-F  308-CHECK-R  ACE1-F  ACE1-R  TEF2-F  TEF2-R  ACE1-TEF2-F  ACE1-TEF2-R  FET3-qPCR-F  FET3-qPCR-R  CRS5-qPCR-F  CRS5-qPCR-R  SOD1-qPCR-F  SOD1-qPCR-R  CUP1-qPCR-F  CUP1-qPCR-R  ACE1-qPCR-F  ACE1-qPCR-R  HES1-qPCR-F  HES1-qPCR-R  ACT1-qPCR-F  ACT1-qPCR-R | Primers for constructing strain MO1 with the deletion of *ADY2*  gaagctcgtcaaaactggacctctattgaaaacatcaaagaattg atctacacttagtagaaattgatcatttatctttcactgcggagaag  atgatcaatttctactaagtgtagat**gtatcttggaagcttacgaa**gctttttttgtt  atgatcaatttctactaagtgtagat**aagccacctctgcaaaacgg**gctttttttgtt  tagaggtccagttttgacgagcttcaaaacgttccttttccttctta  gataggcgtcgtatatagtctcttct  cacctgcatgtggttgtttagttatatctgtgttagc  gtggttgttatgcaggtgttgctacaaagcagaattc  cgcgcatttggatcgtccagcccctg  Primers for deletion of *ACE1*  tcttgcccagtcgtaaggatattatt  tctgtgaatacactgattatcagtcctc  gactgataatcagtgtattcacagatgcatctggacatctatataaatat  gaacaggattctccagtgttggtatt  Primers for constructing strains with overexpression of *HES1*  atgatcaatttctactaagtgtagat**ctacatattgctaccacttc**gctttttttgtt gtgaaaaatacctagacctaatagagaatagtggaaacaacaatggcaaaatatt  tagtggaaacaacaatggcaaaatattgttcaggtacccgcgttaaggggctgcc  cttcgtaaatagtattatattgctatatgttttgcc  catatagcaatataatactatttacgaagatgtctcaacacgcaagctcatcttct  tgtttggcgctgatcgccatgccatgatggtttgacttgtaacttcattggga  Primers for constructing strains with overexpression of *CUP1*  catatagcaatataatactatttacgaagatgttcagcgaattaattaacttccaaa  tgtttggcgctgatcgccatgccatggatcattgaaagtgacggggataacag  Primers for constructing strains with overexpression of *SOD1*  catatagcaatataatactatttacgaagatggttcaagcagtcgcagtgttaaag  tgtttggcgctgatcgccatgccatgctcgtggctttatttttgttggtaagga  atgaatagctttctgtggattttccatattgtttggcgctgatcgccatgccatg  agcatggtatggcgcggaagaaaccg  caaatatcatcatgtgacttatttgtacta  Primers for constructing strain MO3 with overexpression of *ACE1* under *Cit1* promoter  catatagcaatataatactatttacgaagatggtcgtaattaacggggtcaaatatg  tgtttggcgctgatcgccatgccatgaactttaggatggttacgtatcctgtg  Primers for constructing strain MO4 with overexpression of *ACE1* under *TEF2* promoter  tagtggaaacaacaatggcaaaatattaagcgtaggcgcttcccctgccggctg  gtttagttaattatagttcgttgaccgtatattct  atacggtcaacgaactataattaactaaacatggtcgtaattaacggggtcaaatatg  tgtttggcgctgatcgccatgccatgaactttaggatggttacgtatcctgtg  Primers for qPCR  tggtcacggacttgacgaag  ttcgaccacggtcatttcgt  aaatatgtgactgttaaggcga  ctaacatttttcacaattgca  cgagccaaccactgtctctt  aacgacgcttctgcctacaa  tcagcgaattaattaacttcc  cccagagcagcatgacttct  cagacgcaagggaagaccat  cctccctttgatttccggct  cctgattacaaccccgcctt  aaacacctgaccattggcca  gaaatgcaaaccgctgctca  cctgggaacatggtggtacc |  |
