# Supplementary material for: Transcriptome Analysis Reveals a Promotion of Carotenoid Production by Copper Ions in Recombinant Saccharomyces cerevisiae
Source: Microorganisms. 2021 Jan 23;9(2):233. doi: 10.3390/microorganisms9020233 (PMC7912134; doi:10.3390/microorganisms9020233)
Supplement: Supplementary file 1 [file microorganisms-09-00233-s001.zip › Figure S1.docx]

**Supplementary Materials**

**Transcriptome Analysis Reveals a Promotion of Carotenoid Production by Copper Ions in Recombinant *Saccharomyces cerevisiae***

Buli Su, Anzhang Li, Ming-Rong Deng, Honghui Zhu*

State Key Laboratory of Applied Microbiology Southern China, Guangdong Provincial Key Laboratory of Microbial Culture Collection and Application, Guangdong Microbial Culture Collection Center (GDMCC), Guangdong Institute of Microbiology, Guangdong Academy of Sciences, Guangzhou 510070, People’s Republic of China.

*Corresponding author

Honghui Zhu: E-mail: zhuhh@gdim.cn

Tel: +86-020-87137669, Fax: +86-020-87685699

**Figure S1.** Shake-flask fermentations (96 h) of BL03-D-4, MO3, SC106 and MO7 in YPD medium with different yeast extracts, modified YPD medium (YPM) or YPD2 medium supplemented with Zn^2+^(0.08 mM) or/and Cu^2+^(0.08 mM) (A). Normalized spot tests of yeast culture dilutions on different media plates, strains were adjusted to an OD_600_ = 1.0, serially diluted to 1:0, 1:10, 1:100, 1:1000, and then spotted on agar supplemented with different media cultivated for 72 h or 120 h (B, C).


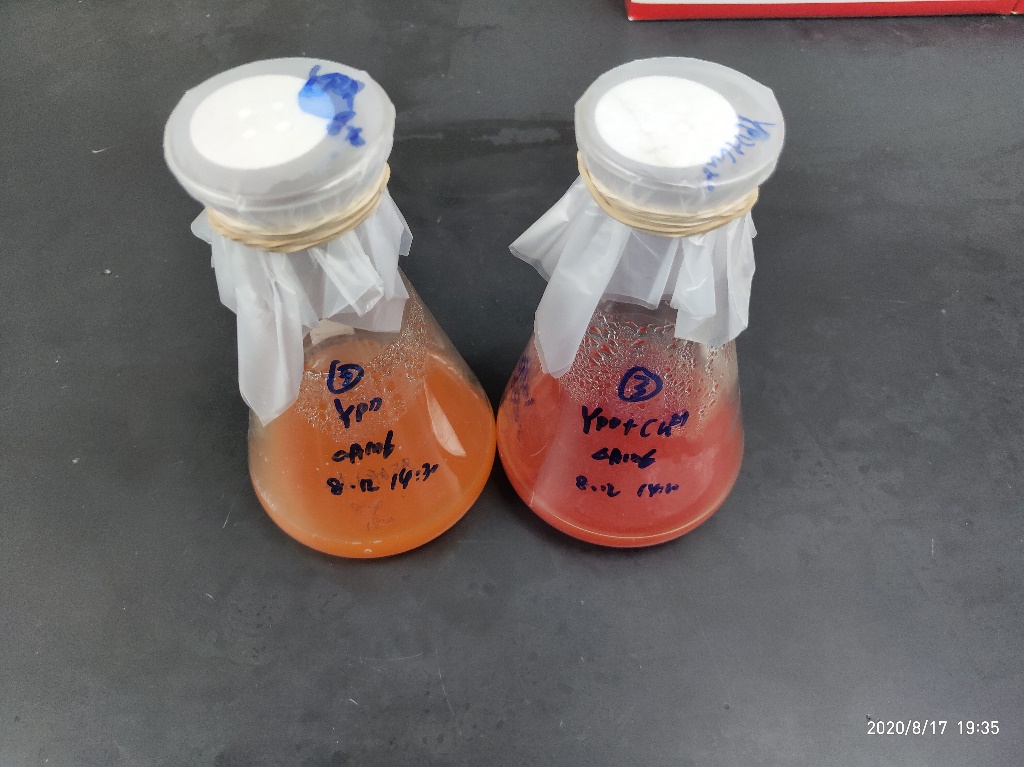

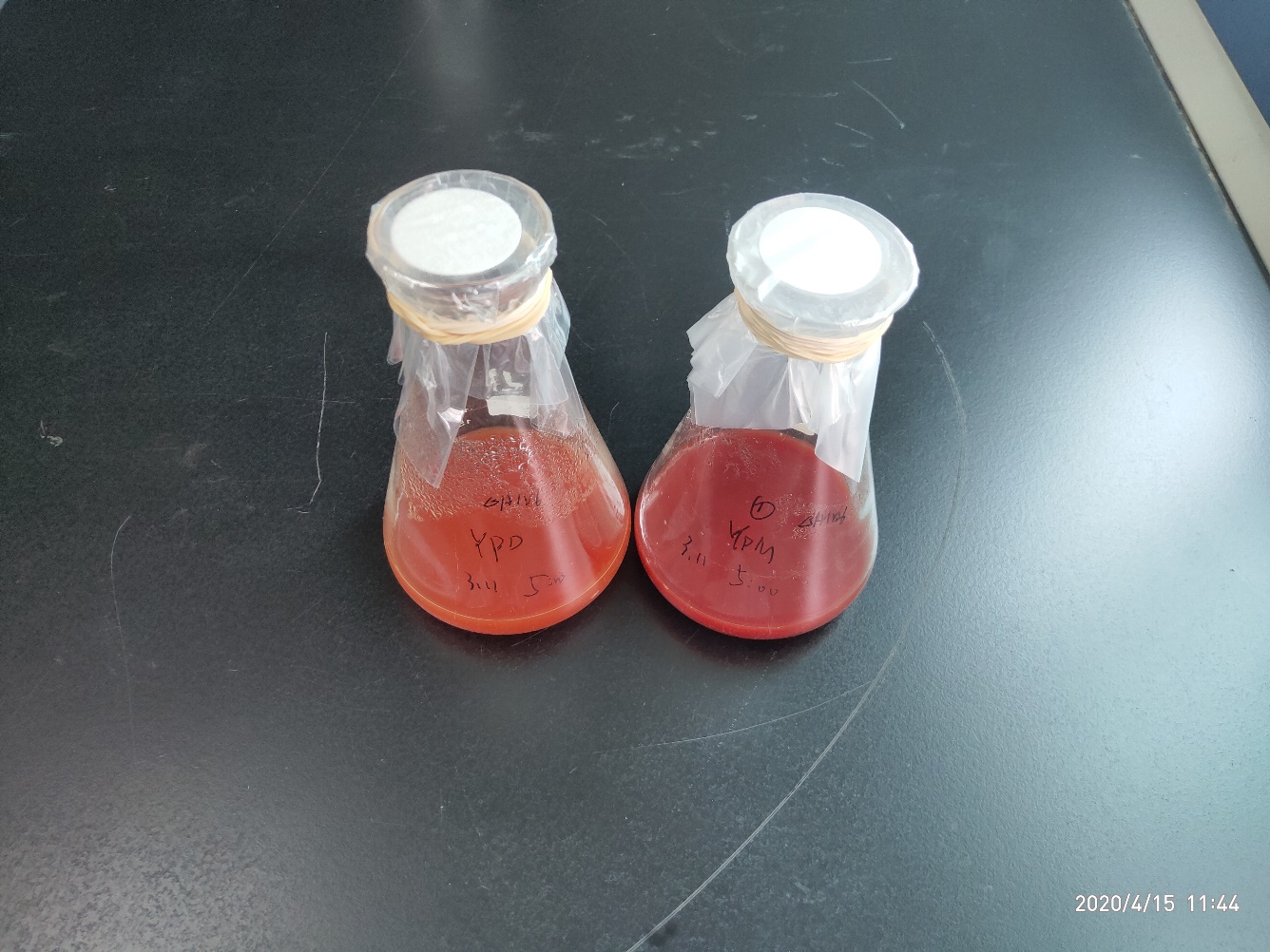


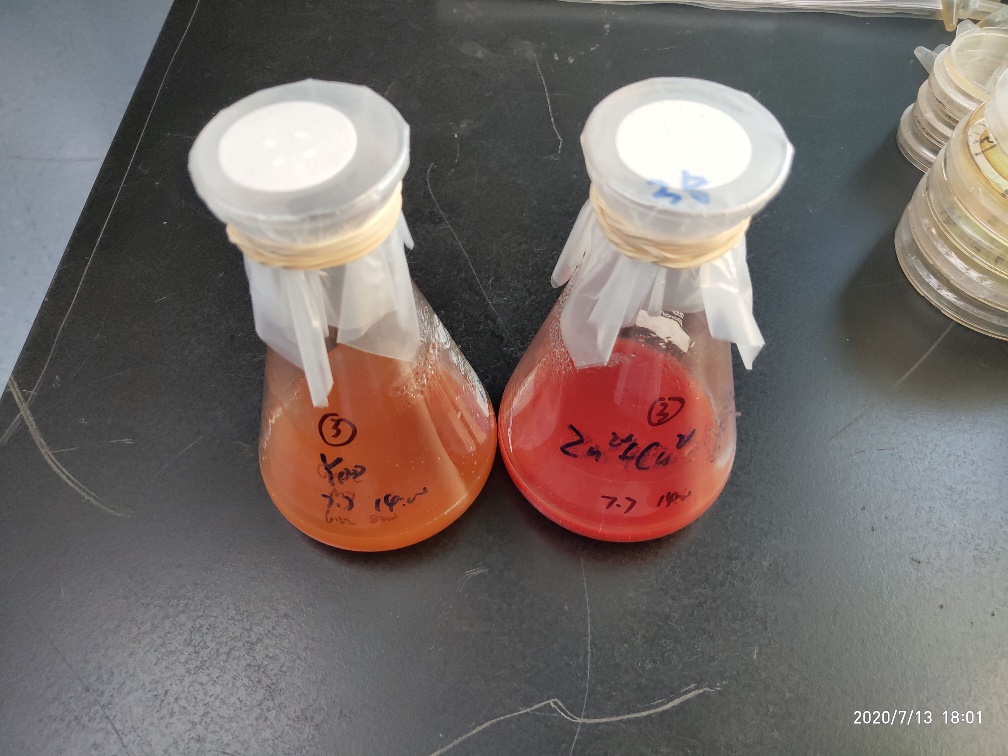

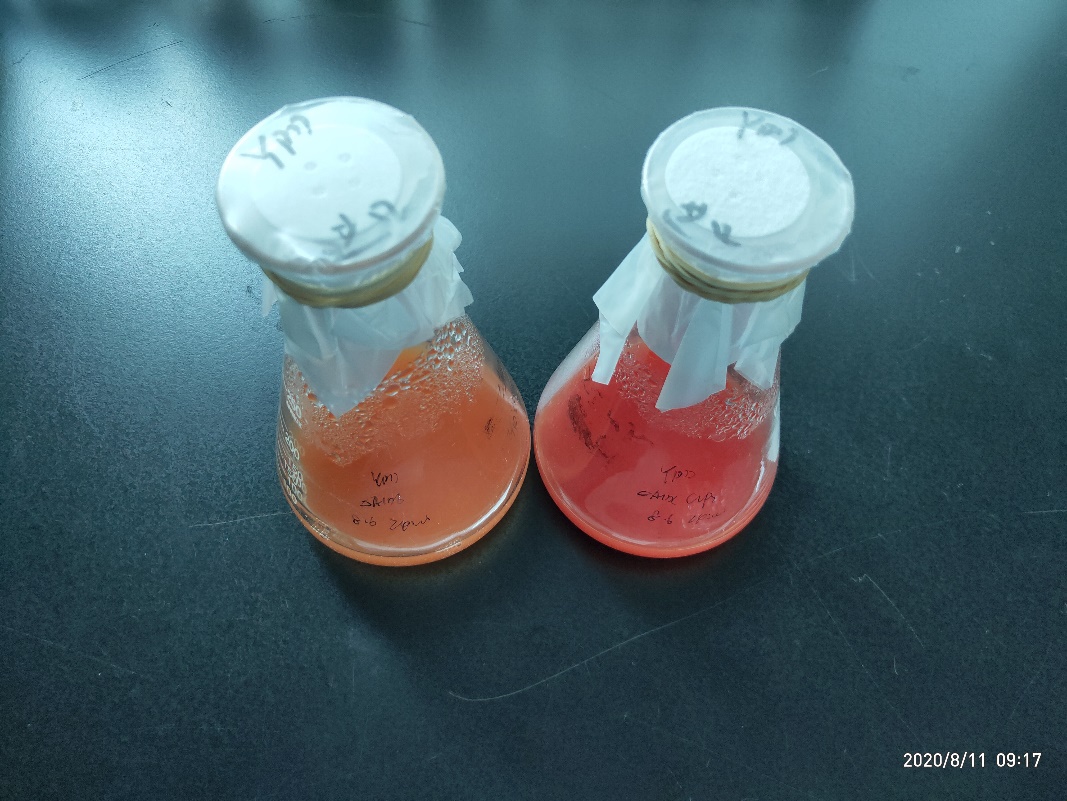
BL03-D-4 in YPD3 YPM YPD2 YPD2+Cu^2+^


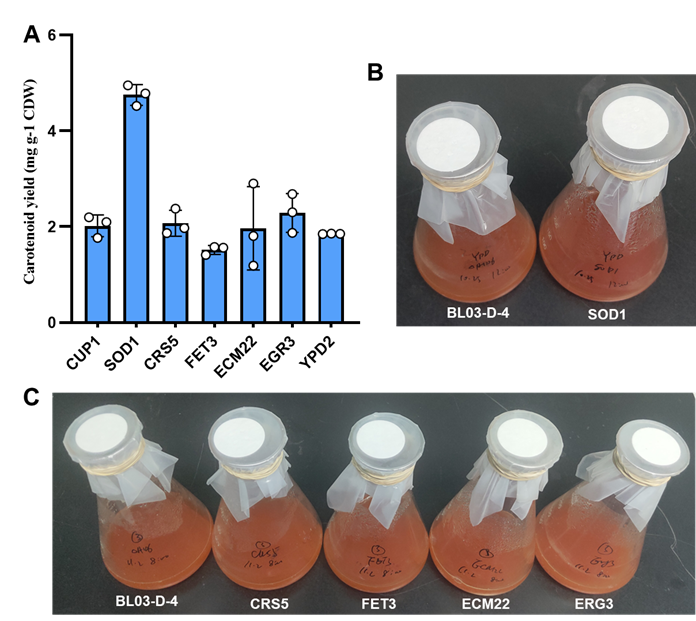

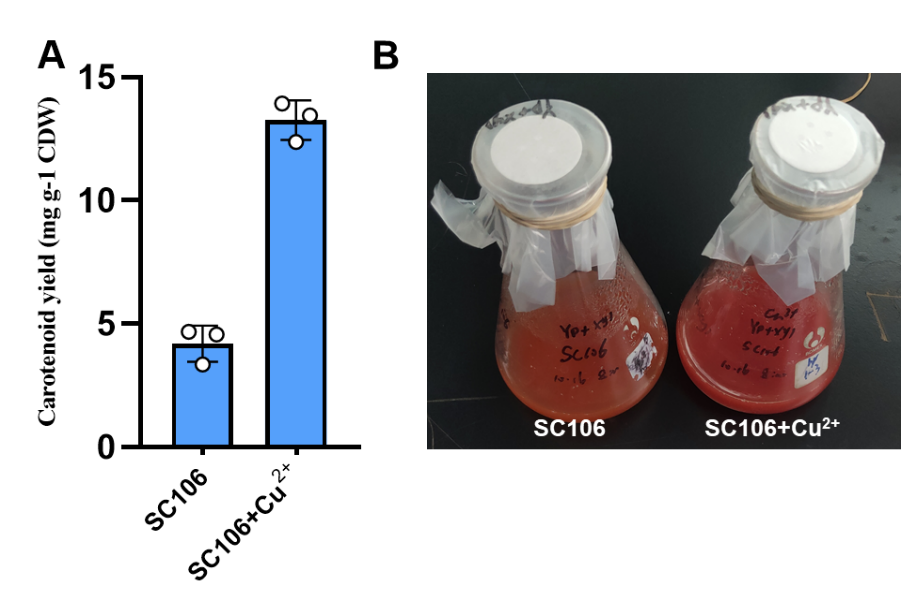
 YPD2 YPD2+Zn^2+^+Cu^2+^ BL03-D-4 in YPD2 MO3 in YPD2

SC106 in YPX YPX+ Cu^2+^ BL03-D-4 in YPD2 MO7 in YPD2

Figure S1A


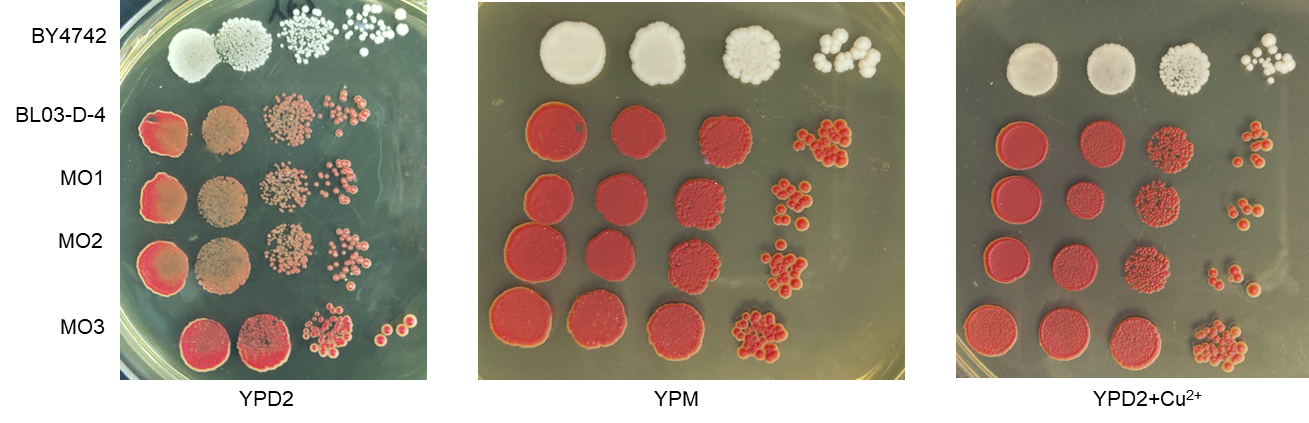


Figure S1B


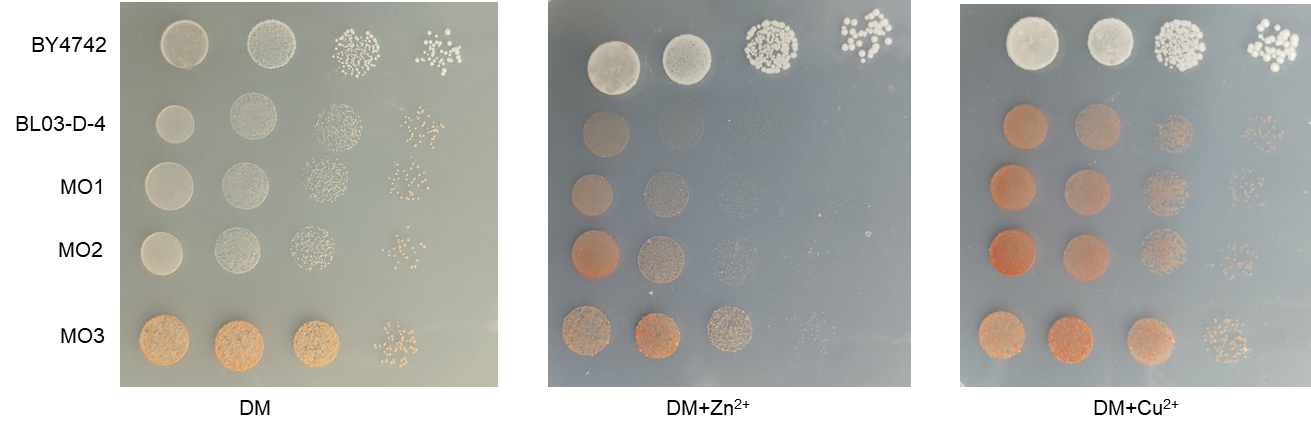


Figure S1C
